# Modulating D-Band Center of SrTiO_3_ by Co Doping for Boosted Peroxymonosulfate (PMS) Activation Under Visible Light

**DOI:** 10.3390/molecules30122618

**Published:** 2025-06-17

**Authors:** Kaining Sun, Xinyi Yang, Fei Qi, Yingjie Liu, Lijing Wang, Bo Feng, Jiankang Yu, Guangbo Che

**Affiliations:** 1 College of Safety Science and Engineering, Liaoning Technical University, Huludao 125105, China; sunkaining02@163.com (K.S.); yang01070321@163.com (X.Y.); 2 Jilin Provincial Key Laboratory of Western Jilin’s Clean Energy, Baicheng Normal University, Baicheng 137000, China; feiqi20060321@163.com (F.Q.); lyj@bcnu.edu.cn (Y.L.); 3 Henan Engineering Center of New Energy Battery Materials, Henan D&A Engineering Center of Advanced Battery Materials, College of Chemistry and Chemical Engineering, Shangqiu Normal University, Shangqiu 476000, China; wanglijing1989@126.com

**Keywords:** photo-fenton reaction, Co-SrTiO_3_, PMS activation, carrier extraction

## Abstract

Peroxymonosulfate (PMS)-based advanced oxidation technology has emerged as an effective means for removing organic pollutants from water due to its strong oxidizing ability. However, enhancing the activation efficiency of PMS represents a key challenge at present. SrTiO_3_, a typical perovskite metal oxide, holds potential in the field of the photocatalytic degradation of pollutants, yet its application is limited by the wide bandgap and fast carrier recombination rates. This study optimized the photocatalytic performance of SrTiO_3_ by regulating its electronic structure and optical properties through cobalt (Co) doping. Experimental results (TRPL, TPV, UV–Vis DRS, ESR, etc.) and DFT calculations (GGA-PBE) demonstrated that Co doping shifted the d-band center of SrTiO_3_ upwards, optimized the adsorption energy of SO_4_^−^, enhanced the sunlight response range, and significantly improved carrier extraction efficiency. Under visible light irradiation, 2,4-dichlorophenol (2,4-DCP) could be effectively degraded within 60 min in a wide pH range. Through Fukui function calculation (B3LYP/6-31G*) and experimental characterization analysis (HPLC-MS and IC), the possible degradation pathways of 2,4-DCP and the mechanism for photocatalysis were investigated. The toxicity analysis (T.E.S.T) confirmed the reduced toxicity of the degradation products of 2,4-DCPs. This study provides a reference for the catalyst design and optimization strategy of PMS-based advanced oxidation technology.

## 1. Introduction

Dichlorophenols (DCPs), as typical persistent organic pollutants (POPs), are widely present in electronic waste treatment solutions, dye wastewaters, and pharmaceutical wastewaters [1,2,3,4]. Their high chemical stability and carcinogenic properties pose a serious threat to the ecological environment, making efficient and environmentally friendly wastewater treatment technologies a crucial area of scientific research and technological application. Among these technologies, Peroxymonosulfate (PMS) advanced oxidation processes have demonstrated great potential for removing organic pollutants in water due to their strong oxidizing properties and efficient degradation capabilities. However, one of the key challenges currently faced is how to further enhance the activation efficiency and degradation rate of PMS [5].

Perovskite oxides are promising photocatalysts due to their low cost, favorable optical properties, and high catalytic activity [6,7,8]. They are stable under harsh conditions because of their flexible crystal structure and the presence of transition metals. Moreover, their structural and physicochemical properties can be easily tailored by adjusting their chemical compositions. This versatility has led to a significant interest in their applications for photocatalytic water remediation [9,10]. SrTiO_3_, as a typical perovskite metal oxide, has attracted extensive attention in the field of the photocatalytic degradation of pollutants due to its strong redox ability and good structural stability [11,12,13,14]. However, the wide bandgap of SrTiO_3_ limits its utilization of visible light, and its rapid carrier recombination rate results in high activation and adsorption energy barriers for ⸱SO_4_^−^ [15,16]. Metal doping is an effective method to regulate the electronic structure and optical properties of SrTiO_3_, thereby optimizing its photocatalytic performance [17,18,19,20]. Notably, the precise modulation of the d-band center position in SrTiO_3_, achievable through the adjustment of dopant type and concentration, directly governs its carrier extraction and transport capabilities [21,22,23,24]. Furthermore, the d-band center of transition metals exhibits a strong correlation with their molecular adsorption affinity [25,26,27,28]. Consequently, regulating this electronic parameter enables the manipulation of both adsorption strength and configuration on the metal surface towards peroxymonosulfate (PMS) and its reaction intermediates, thereby critically influencing PMS activation efficiency. This electronic structure modulation constitutes a highly significant strategy for enhancing photocatalyst performance.

Cobalt (Co), as an abundant transition metal element on Earth, has significant advantages in regulating the electronic structure and catalytic performance of catalysts [19,29,30,31]. Co doping can effectively alter the electronic structure of the host material SrTiO_3_, thereby enhancing the activation efficiency of PMS [32,33,34]. Optimizing the adsorption-activation dynamics of peroxymonosulfate (PMS) through the precise adjustment of cobalt (Co) doping levels facilitates the cleavage of peroxide bonds, thereby generating enhanced concentrations of highly oxidizing species (e.g., sulfate radicals, hydroxyl radicals). This consequently accelerates the degradation kinetics of organic pollutants, such as 2,4-dichlorophenol (2,4-DCP) [35,36,37]. Moreover, Co doping effectively broadens the operational pH range for efficient organic degradation, ensuring sustained high catalytic activity across diverse aqueous matrices [38,39], thus demonstrating significant application potential in critical environmental remediation fields, including wastewater treatment and groundwater purification.

Based on the above discussion, this paper enhances the activation of PMS and the efficient degradation of 2,4-DCP by regulating the d-band center and carrier extraction efficiency of SrTiO_3_ through Co doping. The research results indicate that Co doping shifts the d-band center of SrTiO_3_ up by 0.778 eV (compared to the pure sample), optimizes the PMS adsorption energy to −10.2 eV, increases the carrier extraction efficiency by about six times, and allows for the degradation of 2,4-DCP (30 mg/L) by 88% under visible light within 60 min. Moreover, understanding how material structure governs degradation pathways is key to revealing material properties [40,41]. Thus, density functional theory (DFT) calculations coupled with the HPLC-MS technique were also conducted to explore the degradation pathway of 2,4-DCP. This research may offer a reference to design an efficient and environmentally friendly method for treating refractory organic pollutants.

## 2. Results and Discussion

### 2.1. Structure and Composition Characterization of Photocatalysts

**Figure 1:**
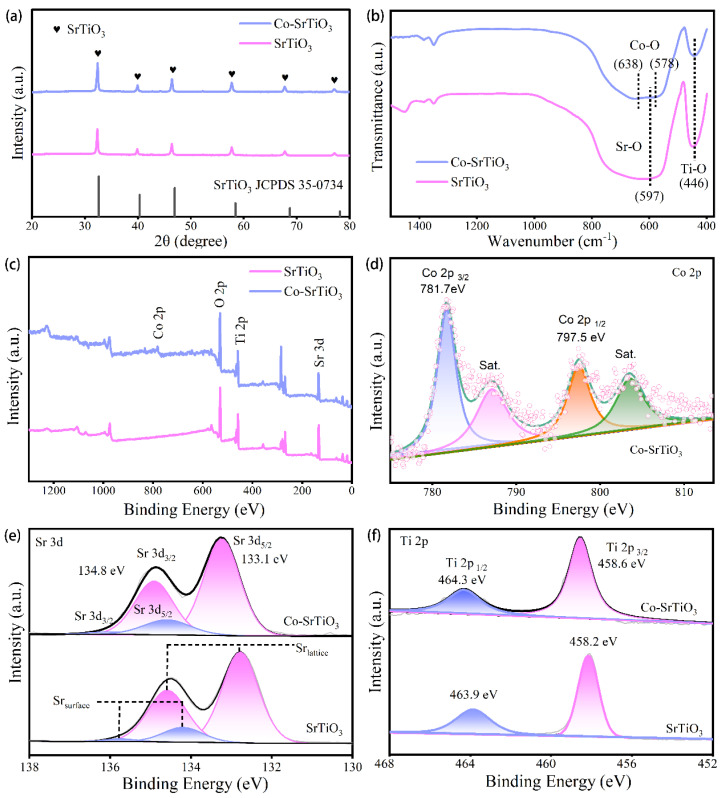
The (**a**) XRD patterns, (**b**) FT-IR spectra, (**c**) XPS survey spectra, (**d**) Co 2p, (**e**) Sr 3d and (**f**) Ti 2p high-resolution XPS spectra for samples.

The XRD patterns of Co-SrTiO_3_, shown in Figure 1a, revealed that Co doping did not alter the crystal structure of SrTiO_3_, as evidenced by the well-matched diffraction peaks of Co-SrTiO_3_ and SrTiO_3_ with the reference data JCPDS 35-0734 [42]. Figure 1b shows the FTIR spectra of SrTiO_3_ and Co-SrTiO_3_. The peaks at 446 and 597 cm^−1^ correspond to the stretching vibrations of Ti-O and Sr-O, respectively [43]. New peaks at 578 and 638 cm^−1^ appear in the infrared spectrum of Co-SrTiO_3_, corresponding to the vibrations of Co-O bonds [44]. To verify Co doping, XPS characterization was performed on SrTiO_3_ and Co-SrTiO_3_ powders, as shown in Figure 1c–f. The XPS survey spectra (Figure 1c) indicated that both SrTiO_3_ and Co-SrTiO_3_ contained Sr, Ti, and O elements, with Co-SrTiO_3_ also containing small amounts of Co. The high-resolution Co 2p XPS spectra of Co-SrTiO_3_ are shown in Figure 1d, revealing two peaks at 781.7 and 797.5 eV, corresponding to Co 2p_3/2_ and Co 2p_1/2_, respectively [45]. In Figure 1e, the fitted peaks at 134.5 and 132.7 eV correspond to Sr 3d_3/2_ and Sr 3d_5/2_ states, respectively [46]. Concurrently, Sr segregation is a characteristic feature commonly observed in SrTiO_3_ materials prepared via solid-state reaction. However, the extent of Sr segregation shows no significant difference between the SrTiO_3_ and Co-SrTiO_3_ samples (Figure 1e). Therefore, this Sr segregation phenomenon is not the primary factor responsible for the observed differences in their photocatalytic performance [47,48]. In Figure 1f, the peaks at 463.9 and 458.2 eV correspond to Ti 2p_1/2_ and Ti 2p_3/2_ states [49]. For SrTiO_3_, the binding energies of Sr and Ti elements shifted to higher values, indicating electron transfer between elements in the SrTiO_3_ lattice after Co doping. These XPS results confirm the successful Co doping upon SrTiO_3_.

**Figure 2:**
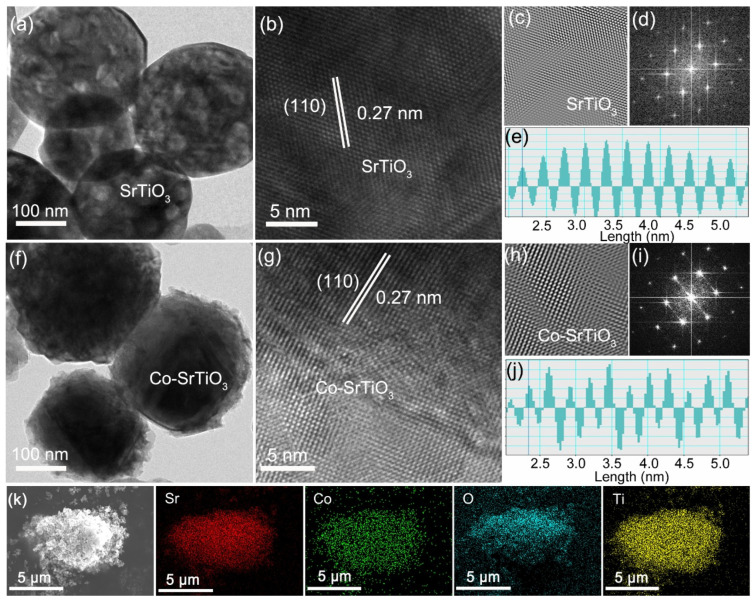
TEM images (**a**), HRTEM and diffraction spot images (**b**–**e**) of SrTiO_3_. TEM images (**f**), HRTEM and diffraction spot images (**g**–**j**) of Co-SrTiO_3_. (**k**) EDS images of Co-SrTiO_3_.

The micromorphology of SrTiO^3^ and Co-SrTiO_3_ was observed using transmission electron microscopy (TEM) and scanning electron microscopy (SEM), as shown in Figure 2 and Figure S1. Both samples exhibit spherical forms (Figure 2a,f), and a lattice fringes of 0.27 nm were observed (Figure 2b,c), corresponding to the (110) crystal plane of SrTiO_3_ [32]. Additionally, the bright diffraction spots of SrTiO_3_ in Figure 2c–e confirmed its crystalline nature, indicating good crystallinity. The diffraction spots of Co-SrTiO_3_ in Figure 2h–j are almost identical to those of SrTiO_3_, suggesting that Co doping did not affect the crystal phase structure of SrTiO_3_. This observation is consistent with the XRD results. The SEM images in Figure S1 show that the particle size of Co-SrTiO_3_ is smaller than that of SrTiO_3_. This may be due to Co doping affecting the nucleation process of SrTiO_3_ and improving its crystal quality, which is consistent with the slightly higher XRD peak intensity of Co-SrTiO_3_ in Figure 1a [17]. Furthermore, the small particle diameter results in a larger specific surface area (Figure S2a). Co doping also induces the formation of pores on the surface of SrTiO_3_, resulting in Co-SrTiO_3_ exhibiting a larger pore size of 64 nm, in addition to the 2–10 nm micropores (Figure S2b and Table S1). This enhances the ability of Co-SrTiO_3_ to adsorb pollutant molecules. As shown in the EDS mapping images (Figure 2k), Co-SrTiO_3_ demonstrates a uniform distribution of elements, including Sr, Co, O, and Ti, indicating that Co has been effectively doped into the SrTiO_3_ crystal structure. EDS analysis (Figure S3) further confirmed the presence of Co, Ti, Sr, and O elements in Co-SrTiO_3_.

### 2.2. Photocatalytic Performance and Durability

**Figure 3:**
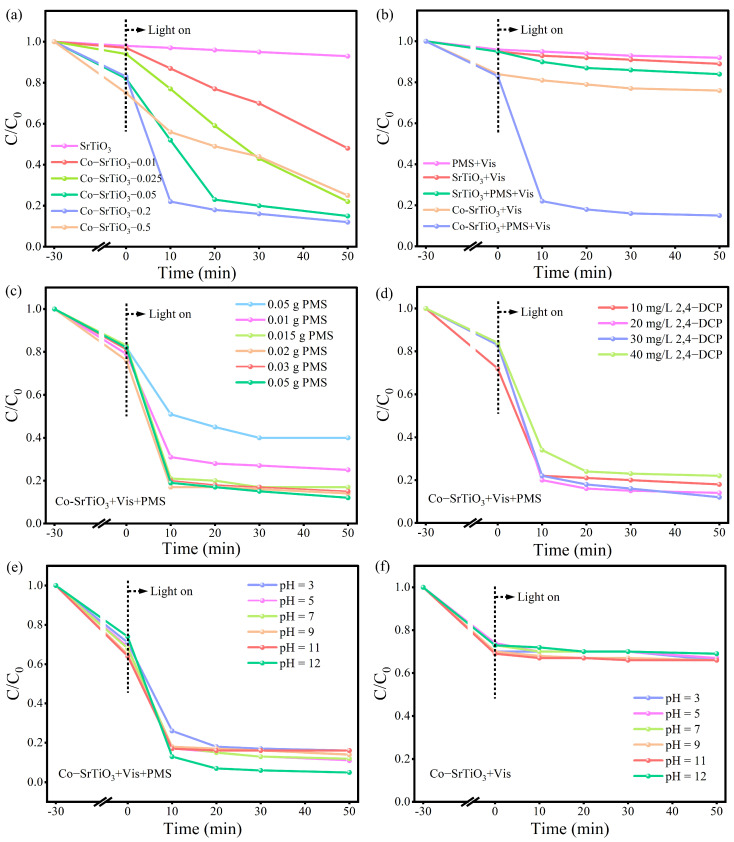
(**a**) Photocatalytic degradation curves of 2,4DCP (30 mg/L, pH = 5) over SrTiO_3_ and Co-SrTiO_3_-X with PMS (0.015 g). (**b**) Comparison of 2,4-DCP photodegradation (30 mg/L, pH = 5) over SrTiO_3_ and Co-SrTiO_3_-0.2 with and without PMS (0.015 g). (**c**) Comparison of 2,4-DCP (30 mg/L, pH = 5) photodegradation over Co-SrTiO_3_-0.2 with different amounts of PMS. (**d**) Photocatalytic degradation curves of different 2,4-DCP concentrations over Co-SrTiO_3_-0.2 (pH = 5) with PMS (0.015 g). Photodegradation efficiency curves of 2,4-DCP (30 mg/L) under different pH conditions with (**e**) and without (**f**) PMS (0.015 g).

To optimize the cobalt doping dosage in SrTiO_3_, pure SrTiO_3_ and Co-SrTiO_3_-X samples (where X = 0.01, 0.025, 0.05, 0.2, and 0.5) were employed to activate peroxymonosulfate (PMS) for the degradation of a 2,4-dichlorophenol (2,4-DCP) solution at a concentration of 30 mg/L, as illustrated in Figure 3a. After 30 min of stirring in the dark, Co-SrTiO_3_-0.5 demonstrated the highest adsorption capacity for the 2,4-DCP contaminant. Moreover, a comparison of the adsorption properties between SrTiO_3_ and Co-SrTiO_3_-X reveals that with increasing cobalt doping levels, the adsorption performance of the corresponding samples improves. This indicates that Co doping enhances the adsorption of contaminants on the surface of strontium titanate, thereby contributing to the enhancement of photocatalytic performance. Meanwhile, photocatalysts with varying amounts of Co doping exhibit different degradation efficiencies under visible light irradiation. The degradation efficiencies of Co-SrTiO_3_-0.01, Co-SrTiO_3_-0.025, Co-SrTiO_3_-0.05, Co-SrTiO_3_-0.2, and Co-SrTiO_3_-0.5 for 2,4-Dichlorophenol (2,4-DCP) are 52%, 78%, 85%, 88%, and 75%, respectively. Notably, Co-SrTiO_3_-0.2 (hereinafter referred to as Co-SrTiO_3_) demonstrates the highest photocatalytic degradation efficiency of 2,4-DCP, achieving a performance that is 12.6 times greater than that of pure SrTiO_3_.

As shown in Figure 3b, the photocatalytic efficiency of Co-doped SrTiO_3_ combined with PMS activation is significantly higher than that without PMS activation. In contrast, the photocatalytic degradation performance of pure SrTiO_3_, both alone and in combination with PMS activation, is considerably lower than that of Co-doped SrTiO_3_. Moreover, pure SrTiO_3_ and PMS exhibit minimal photocatalytic activity under light irradiation. These results demonstrate that Co-doping significantly enhances PMS activation for pollutant degradation under visible light.

Considering the economic benefits and substrate removal, we investigated the effects of varying dosages of PMS and pollutant concentrations on the photocatalytic efficiency of Co-SrTiO_3_ in degrading 2,4-DCP. As the amount of PMS increased (Figure 3c), more active species were generated, resulting in a gradual improvement in degradation efficiency. However, when the PMS dosage exceeded 0.015 g, the removal efficiency for 2,4-DCP did not show a significant enhancement. This may be attributed to the quenching reaction between excess PMS (HSO_5_^−^) and free radicals (·SO_4_^−^ and ·OH), leading to a reduction in degradation activity [50]. Therefore, in subsequent experiments, the PMS dosage was maintained at 0.015 g. As illustrated in Figure 3d, the Co-SrTiO_3_ photocatalytic PMS activation system is capable of adapting to a wide concentration range of 2,4-DCP. When the concentration of 2,4-DCP solution is increased to 40 mg/L, the degradation rate of 2,4-DCP by Co-SrTiO_3_ can still exceed 75% within 50 min.

The activity of advanced oxidation processes (AOPs) is strongly dependent on the pH value of the reaction system. Therefore, it is crucial to evaluate the performance of photocatalytic materials across a wide pH range. Figure 3e illustrates the effect of various pH conditions on the removal efficiency of 2,4-DCP in the Co-SrTiO_3_ system. Co-SrTiO_3_ exhibited excellent photocatalytic performance (remaining above 80%) across a wide pH range (pH = 3, 5, 7, 9, 11, 12), with the highest catalytic efficiency observed at pH = 12. However, without the addition of PMS, the photocatalytic degradation efficiency of Co-SrTiO_3_ in all pH ranges is only less than 40%. This indicates that Co-SrTiO_3_ possesses good pH universality in acidic, neutral, and alkaline conditions, highlighting its potential for wastewater treatment in complex pH environments. However, in the absence of PMS, the photocatalytic degradation efficiency of Co-SrTiO_3_ across all pH ranges remains below 40%. This highlights the significant impact of Co doping in enhancing the PMS activation performance of SrTiO_3_ for pollutant degradation.

Recognizing that photocatalytic reusability and stability constitute critical performance metrics for wastewater treatment applications, recycling experiments were conducted to quantitatively evaluate these properties of Co-SrTiO_3_. As shown in Figure S4a, after five cycles of photocatalytic degradation experiments, the photocatalytic performance of Co-SrTiO_3_ remained nearly unchanged, with the removal rate of 2,4-DCP consistently around 80%. This indicates that Co-SrTiO_3_ possesses excellent chemical stability and reusability. To further demonstrate the recycling stability of Co-SrTiO_3_, the XRD data of the fresh and used Co-SrTiO_3_ have been provided for comparison (Figure S4b). No phase decomposition or lattice distortion was observed. The retention of identical XRD patterns following five photocatalytic cycles under extended irradiation (>4 h) (Figure S4b) provides definitive evidence for the photothermal stability of Co-SrTiO_3_. This structural robustness, coupled with uncompromised performance, underscores its viability for industrial implementation.

### 2.3. Photo-Electrochemical Properties

**Figure 4:**
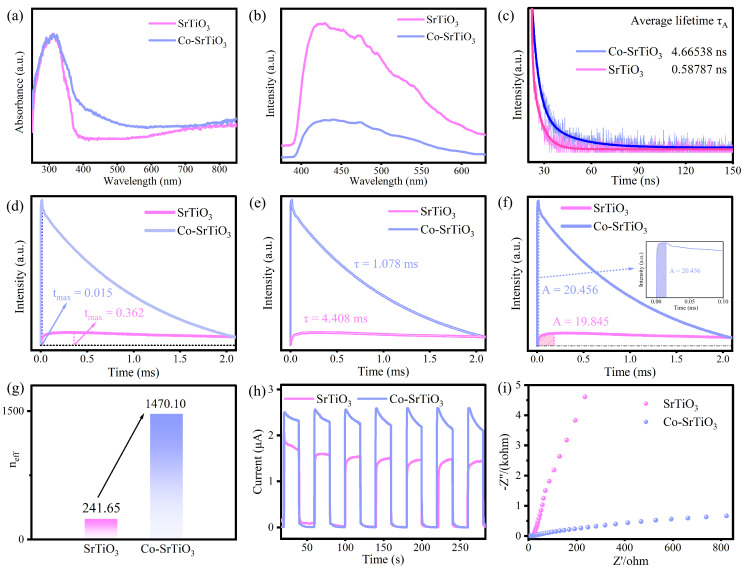
(**a**) UV–Vis DRS, (**b**) steady-state, and (**c**) time-resolved PL spectra of SrTiO_3_ and Co-SrTiO_3_. (**d**) The maximum charge extraction time (t_max_), (**e**) electron decay constants (*τ*), (**f**) amount of charge extraction (A), and (**g**) the number of effective charges (neff) of SrTiO_3_ and Co-SrTiO_3_ estimated from transient photovoltage (TPV) spectra. (**h**) Photocurrent response, and (**i**) EIS plots of SrTiO_3_ and Co-SrTiO_3_.

The UV–Vis DRS data (Figure 4a) indicate that Co-doped SrTiO_3_ exhibits a significantly higher spectral response to visible light in the range of 400–700 nm compared to undoped SrTiO_3_. This finding suggests that cobalt doping effectively modifies the band structure of SrTiO_3_, enhances its absorption of visible light, and increases the generation of photogenerated charge carriers. To investigate the effect of Co doping on the photogenerated carrier separation efficiency of SrTiO_3_, we conducted steady-state and time-resolved photoluminescence (PL) spectra measurements. As illustrated in Figure 4b, the fluorescence intensity of Co-doped SrTiO_3_ is significantly lower than that of undoped SrTiO_3_. A higher PL intensity correlates with a faster carrier recombination rate, indicating that Co doping effectively inhibits the photogenerated electron-hole recombination in SrTiO_3_. Additionally, as demonstrated in Figure 4c, the carrier lifetime of Co-SrTiO_3_ is significantly longer—approximately eight times that of pure SrTiO_3_. The fitting parameters can be found in Table S2. This observation aligns with previous reports concerning Al doping in SrTiO_3_ [17]. Consequently, it can be concluded that Co doping reduces the n-type behavior of SrTiO_3_ and, in turn, extends the charge carrier lifetime and diffusion length. Generally, a longer carrier lifetime in photocatalytic materials is associated with a higher efficiency in the separation of photoelectrons and holes, which leads to enhanced photocatalytic activity. Therefore, the results from the time-resolved photoluminescence (PL) spectra further indicate that Co doping improves the charge separation capability of SrTiO_3_.

Transient photovoltage (TPV) measurements were performed to estimate the maximum charge extraction time (t_max_), the amount of charge extraction (A), and the attenuation coefficient of the photocharge over time (τ) for SrTiO_3_ and Co-SrTiO_3_ [15,51]. The results are shown in Figure 4d–f. Notably, Co-SrTiO_3_ exhibits a shorter t_max_ (0.015 ms), suggesting that Co doping enhances charge transfer efficiency in SrTiO_3_. Furthermore, the charge decay constant (τ) of Co-SrTiO_3_ is smaller compared to that of SrTiO_3_, indicating that Co doping increases the number of active sites and shortens charge transport distances in SrTiO_3_. Additionally, Co-SrTiO_3_ demonstrates a higher amount of extracted electrons (A) than SrTiO_3_, implying a superior ability to generate photocarriers. Consequently, the surface effective electron number (n_eff_) for SrTiO_3_ and Co-SrTiO_3_ can be calculated using Equation (1), with the results illustrated in Figure 4g. It is evident that the effective charge of Co-SrTiO_3_ (1470.10) is approximately six times greater than that of SrTiO_3_ (241.65). Therefore, Co doping significantly improves the rapid extraction and transfer of substantial charges from the SrTiO_3_ surface to PMS, which is crucial for effectively degrading pollutants.

(1) $$n_{eff}=A\times \tau /t_{max}$$

The photo-generated carrier separation–migration behavior of SrTiO_3_ and Co-SrTiO_3_ was further investigated by photocurrent and electrochemical impedance spectroscopy (EIS). It can be observed from Figure 4h that the photocurrent density of Co-SrTiO_3_ is higher than that of SrTiO_3_, indicating a greater efficiency in the separation of photogenerated carriers. This finding aligns with the longer lifetimes of photogenic carriers observed in the time-resolved PL spectra (Figure 4c). Additionally, rapid charge transfer further enhances charge separation. The results of electrochemical impedance spectroscopy (Figure 4i) demonstrate that Co-SrTiO_3_ exhibits the smallest arc radius, signifying the lowest charge-transfer resistance, which is advantageous for the transfer and diffusion of charge carriers in Co-SrTiO_3_.

### 2.4. Reaction Mechanism

**Figure 5:**
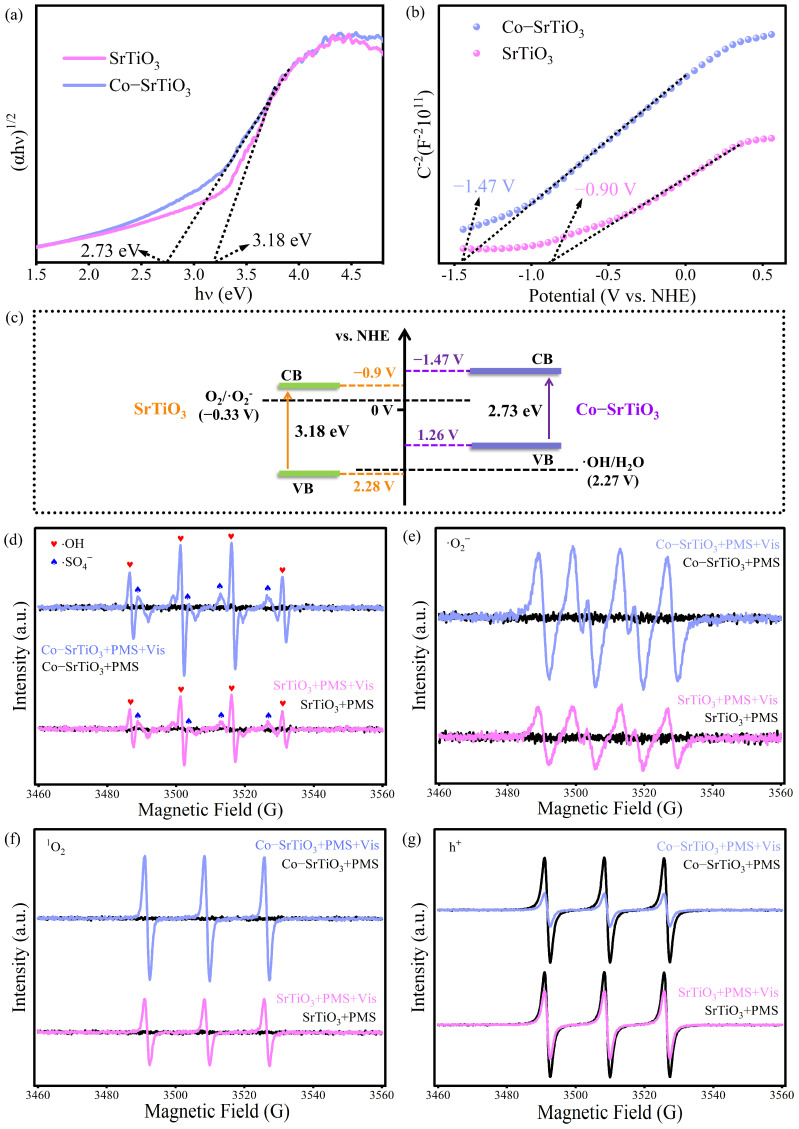
(**a**) Tauc plots derived from UV–Vis DRS data, and (**b**) Mott–Schottky curves of SrTiO_3_ and Co-SrTiO_3_. (**c**) Band structures of SrTiO_3_ and Co-SrTiO_3_. (**d**–**g**) ESR spectra of SrTiO_3_ and Co-SrTiO_3_ under different experimental conditions showing intensities for ·SO_4_^−^,·OH, ·O_2_^−^, ^1^O_2_ radicals, and holes (h^+^).

Based on the UV–Vis DRS test data, the bandgap (E_g_) of SrTiO_3_ and Co-SrTiO_3_ was calculated using Equation (2) proposed by Tauc, where the integer n, which depends on the transition characteristics of the semiconductor, is 2 for indirect transition here [52]. The Tauc plot, shown in Figure 5a, indicates that the band gaps of SrTiO_3_ and Co-SrTiO_3_ are 3.18 eV and 2.73 eV, respectively, determined by extending a tangent along the curve. It is evident that Co doping reduces the bandgap of SrTiO_3_ and broadens the light absorption range. For the conduction position, Mott–Schottky curves were tested, as shown in Figure 5b. The conduction band (E_CB_) values of SrTiO_3_ and Co-SrTiO_3_ were found to be −0.90 and −1.47 V, respectively. Both materials exhibit negative slopes, indicating that they are n-type semiconductors. Using the equation *E_VB_* = *E_CB_* + *E_g_*, we can calculate the valence positions of SrTiO_3_ and Co-SrTiO_3_ to be 2.28 and 1.26 V, respectively. Therefore, we can illustrate the band structure of both materials as shown in Figure 5c. Co-SrTiO_3_ cannot generate OH radicals in water effectively because its valence band position does not reach the redox potential of ·OH/H_2_O (2.72 V) when using holes (h^+^) alone in photocatalytic reactions [53]. Therefore, in the Co-SrTiO_3_ + PMS + visible light system, any ·OH generation is likely to come primarily from the decomposition of PMS. Additionally, the conduction band of Co-SrTiO_3_ has reached the REDOX potential of O_2_/·O_2_^−^ (−0.33 V), allowing it to reduce dissolved oxygen molecules to superoxide free radicals during photocatalytic reactions using photogenerated electrons [54].

(2) $${\left(\alpha h\nu \right)}^{1/n}=C\left(h\nu -E_{g}\right)$$

The electron paramagnetic resonance (EPR) technique was employed to investigate the types of reactive oxygen species (ROS) involved in PMS activation. As shown in Figure 5d, no signals for ·SO_4_^−^ and ·OH were observed in the SrTiO_3_+PMS and Co-SrTiO_3_+PMS systems (in the dark). Upon illumination, distinct ·SO_4_^−^ and ·OH signals emerged in the SrTiO_3_ + PMS + Vis and Co-SrTiO_3_ + PMS + Vis systems. Furthermore, the Co-SrTiO_3_ + PMS + Vis system exhibited stronger signal intensity. This indicates that ·SO_4_^−^ and ·OH are primarily generated from the photocatalytic activation of PMS, and Co doping significantly enhances the activation performance of SrTiO_3_. Similarly, strong ·O_2_^−^ and ^1^O_2_ radical signals were also detected in the illuminated systems, with the Co-SrTiO_3_ system showing a stronger signal (Figure 5e–f). This demonstrates that Co doping also improves the ability of SrTiO_3_ to activate PMS and produce ·O_2_^−^ radicals under visible light. Since holes do not produce ESR response signals, the ESR signals of the trapping agent TEMPO are used to confirm the presence of holes [38]. As shown in Figure 5g, the ESR signal strength is strong under dark conditions due to the absence of hole generation. Upon light irradiation, both SrTiO_3_ and Co-SrTiO_3_ exhibit h^+^ signals; however, Co-SrTiO_3_ shows a weaker h^+^ signal. This is attributed to Co doping, which enhances PMS activation and improves photocatalytic activity. In addition, previous studies indicate that metal doping promotes Sr segregation from the lattice, thereby increasing O vacancy concentration in SrTiO_3_, and the potential presence of O vacancies is expected to influence the photocatalytic activity of SrTiO_3_ [55]. We conducted additional electron spin resonance (ESR) spectroscopy to probe the presence and concentration of surface oxygen vacancies. As shown in Figure S5, both SrTiO_3_ and Co-SrTiO_3_ exhibit characteristic ESR signals at g = 2.003, which is attributed to O vacancies, indicating both SrTiO_3_ and Co-SrTiO_3_ host oxygen vacancies. Critically, the ESR intensity of Co-SrTiO_3_ is slightly stronger than that of undoped SrTiO_3_, indicating a higher concentration of O vacancies in the Co-doped sample. We attribute this to Co doping, which facilitates the partial segregation of Sr from the lattice, creating additional vacancy sites to maintain charge balance [56]. These oxygen vacancies, as electron trapping centers, positively impact the photocatalytic performance of Co-SrTiO_3_, effectively suppressing the recombination of photogenerated carriers. This is consistent with the high performance of Co-SrTiO_3_ shown in Figure 3.

**Figure 6:**
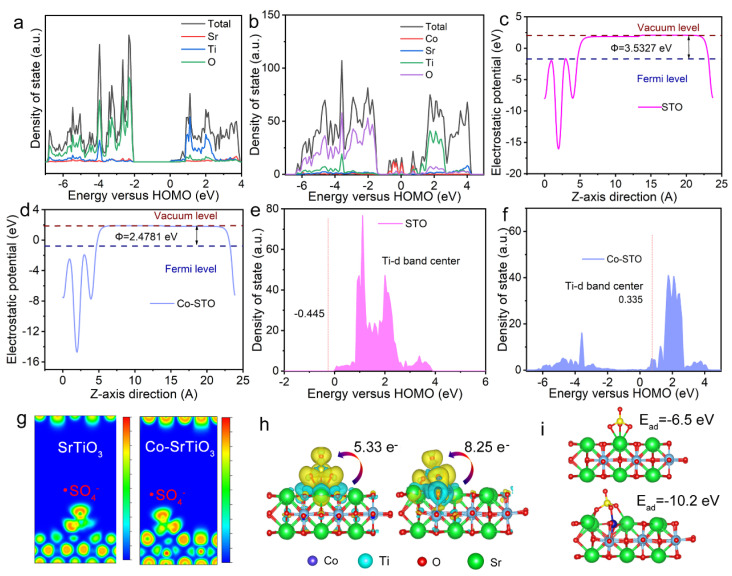
(**a**,**b**) Density of states of SrTiO_3_ and Co-SrTiO_3_, (**c**,**d**) work functions of SrTiO_3_ and Co-SrTiO_3_, (**e**,**f**) d band centers of SrTiO_3_ and Co-SrTiO_3_, (**g**) electron localization function (ELF) images of ·SO_4_^−^ on SrTiO_3_ and Co-SrTiO_3_. (**h**) Bader charges, and (**i**) the adsorption properties of ⸱SO_4_^−^ on SrTiO_3_ and Co-SrTiO_3_.

The significant impacts and optimizations of Co doping on the electronic structure, band gap, work functions, d-band center, as well as the adsorption and activation capabilities of ·SO_4_^−^ on SrTiO_3_ were discussed through density functional theory calculations. Although DFT calculations at the GGA-PBE level typically yield overestimated bandgap results, a comparative analysis between SrTiO_3_ and Co-SrTiO_3_ can still provide reliable qualitative conclusions. Firstly, as evident from Figure 6a,b, compared to undoped SrTiO_3_, Co-SrTiO_3_ exhibits notable changes in its band gap structure. The band gap, representing the energy difference between the valence band maximum and conduction band minimum, determines the material’s light absorption and emission properties [57]. Upon Co doping, it is observed that Co-SrTiO_3_ possesses a narrower band gap, which facilitates electron transitions between energy bands, thereby enhancing the material’s electrical conductivity and solar light utilization efficiency [58,59,60]. This is consistent with the UV–Vis DRS results. Additionally, changes in work function values constitute another crucial impact of Co doping. The work function describes the energy required for electrons to escape from the material’s surface [61,62]. Figure 6c,d compares the work function values of Co-SrTiO_3_ with that of SrTiO_3_, revealing a decrease in the work function of Co-SrTiO_3_. This implies that electrons more easily escape from the Co-SrTiO_3_ surface, favoring charge exchange with external species, such as ·SO_4_^−^, and subsequently promoting the adsorption and activation of ·SO_4_^−^. Furthermore, as shown in Figure 6e,f, Co doping modifies the d-band center of Ti metal. The d-band center is a crucial parameter describing the energy state of the d-band of metal atoms on the material’s surface [63,64]. By comparing the d-band center positions of Co-SrTiO_3_ and SrTiO_3_, it is found that Co doping shifts the d-band center of Ti metal up by 0.778 eV. This upward shift favors the adsorption and activation of species like ·SO_4_^−^ on the material’s surface, as changes in the d-band center alter the interaction strength between metal atoms and anions, thereby optimizing adsorption effectiveness and activation capability [65,66,67]. Figure 6g,h presents electron localization function and Bader charge images, visually demonstrating the impact of Co doping on charge distribution through different color coding (blue representing hole accumulation and yellow representing electron accumulation regions). These images reveal that after Co doping, the charge distribution within the material becomes more uniform and dense, with the Bader charge transfer data increasing from 5.33 e^−^ to 8.25 e^−^, which is beneficial for the adsorption and charge exchange of external species such as ·SO_4_^−^. Finally, Figure 6i shows that under the same conditions, Co-SrTiO_3_ exhibits significantly higher adsorption capacity for ·SO_4_^−^ compared to SrTiO_3_ (from −6.5 eV to −10.2 eV), thereby significantly enhancing the degradation performance of DCPs. The above critical changes brought by Co doping, such as the rise in the Fermi level and the d-band center of titanium (Ti), along with the enhanced adsorption capacity and electron delocalization of ·SO_4_^−^ on the surface of SrTiO_3_, provide a comprehensive explanation for the remarkable photocatalytic activity of Co-SrTiO_3_. These findings are further supported by results from the ESR test.

**Figure 7:**
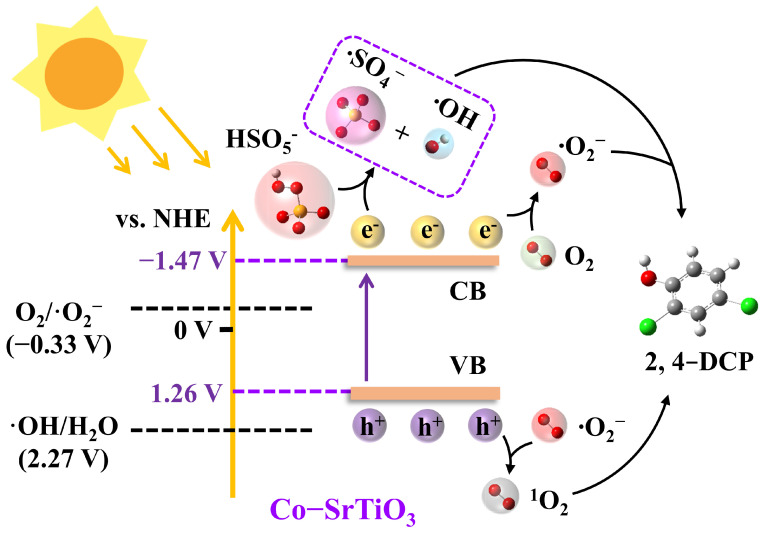
Mechanism of 2,4-DCP degradation in Co-SrTiO_3_+PMS+Vis system.

Based on the results presented, the reaction mechanism for the degradation of 2,4-DCP through the photocatalytic activation of PMS on Co-SrTiO_3_ was proposed, as illustrated in Figure 7. When Co-SrTiO_3_ absorbs energy from photons of a specific wavelength, the photogenerated electrons leap from the valence band (VB) to the conduction band (CB). The PMS (HSO_5_^−^) adsorbed on the surface of Co-SrTiO_3_ would be activated, breaking down into hydroxyl radicals (∙OH) and sulfate radicals (SO_4_^−^). At the same time, the electrons present on the highly reductive CB can effectively reduce dissolved O_2_, generating ∙O_2_^−^ radicals. Furthermore, the holes present on VB can further oxidize the ∙O_2_^−^ radical into ^1^O_2_. These active species, identified through ESR testing, energetically attack 2,4-DCP molecules, leading to their compelling degradation into smaller, less harmful fragments. The possible reaction equations are listed below [68,69]:

Co-SrTiO_3_ + Vis→e^−^ + h^+^ (3)

e^−^ + O_2_→⸱O_2_^−^ (4)

h^+^ + ⸱O_2_^−^→^1^O_2_ (5)

e^−^ + HSO_5_^−^→⸱SO_4_^−^ + OH^−^ (6)

e^−^ + HSO_5_^−^→SO_4_^2−^ + ⸱OH (7)

⸱SO_4_^−^/⸱OH/h^+^/^1^O_2_/⸱O_2_^−^ + 2,4-DCP→Products (8)

### 2.5. Reaction Pathways and Toxicity of Products

**Figure 8:**
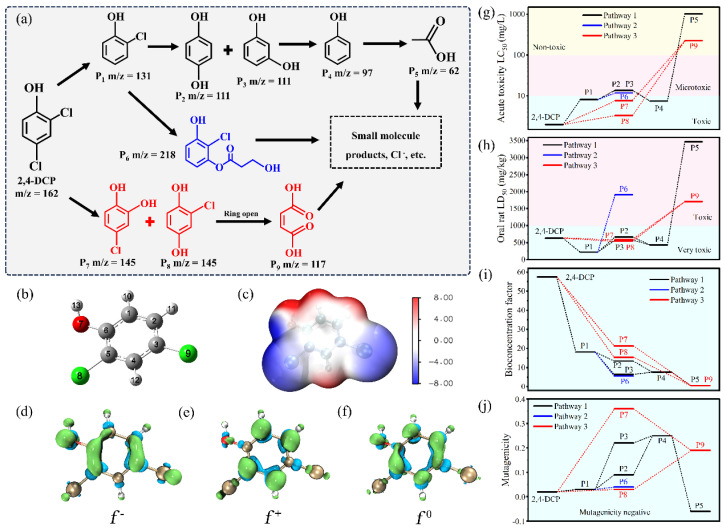
(**a**) The reaction pathways of 2,4-DCP in Co-SrTiO_3_ + PMS + Vis system. (**b**) The structure and atom number, (**c**) the surface electrostatic potential (SEP) of 2,4-DCP. (**d**–**f**) The visible schematic of *f*^+^, *f*^−^, and *f*^0^ values from the Fukui function calculation of 2,4-DCP. (**g**) Acute toxicity LC_50_ for Daphnia magna, (**h**) oral rat LD_50_, (**i**) bioaccumulation factor, and (**j**) mutagenicity of 2,4-DCP and photodegradation intermediates of 2,4-DCP calculated using T.E.S.T. software (version 5.1).

LC-MS was used to identify the degradation intermediates of 2,4-DCP after 10 min of photocatalysis, with the results shown in Figure S6. Based on these findings, three degradation pathways for 2,4-DCP were established (Figure 8a). Initially, the C-Cl bonds in the 2,4-DCP molecule are attacked by active species, such as ∙O_2_^−^, and ∙OH, leading to dechlorination and the formation of products P1 (*m*/*z* = 131), P7, and P8 (*m*/*z* = 145) [2,35,58]. Products P2 and P3 are generated from the second stage of desulfurization of P1, which then undergoes further decomposition into smaller molecules through dehydroxylation and ring-opening reactions. At the same time, product P1 can produce P6 when it is attacked by ∙O_2_^−^ radicals and holes (h^+^). Products P7 and P8 are degraded to form P9 through a ring-opening reaction. Ultimately, products P5, P6, and P9 break down into smaller molecular products and Cl^−^.

To further analyze the degradation pathway of 2,4-DCP, we calculated the electrostatic potential (ESP) and Fukui functions. In the ESP plot (Figure 8b), which is based on the optimized molecular structure shown in Figure 8a, the blue and red regions indicate electron-rich and electron-deficient areas, respectively. The electron-rich regions (blue) are susceptible to electrophilic attacks, while the electron-deficient regions (red) are susceptible to nucleophilic attacks. Notably, the deepest blue regions appear near the two chlorine (Cl) atoms, suggesting that these are the most vulnerable sites for attacks by electrophilic species. This is consistent with the discovery of dechlorination products (P1, P2, P3, P7, and P8) in HPLC-MS results. Additionally, the Fukui function was also utilized to predict the reaction sites of dichlorophenol molecules. The calculated values for the nucleophilic index (*f*^+^), electrophilic index (*f*^−^), and free radical attack index (*f*^0^) are summarized in Table S3. The ∙O_2_^−^ tends to attack sites with higher *f*^+^ values, while h^+^ prefers sites with higher *f*—values. Furthermore, ∙OH is likely to attack not only sites with higher *f*—values but also those with *f*^0^ values [2,70]. The calculation results indicate that the chlorine (Cl) atoms, as well as carbon atoms C_4_ and C_5_, are the primary sites for the attack of active species. This finding is consistent with the results obtained from ESP analysis and the detection of the ring-opening reaction product P9 through HPLC-MS. To conduct a preliminary evaluation of the safety of the Co-SrTiO_3_ + PMS + Vis system for the removal of dichlorophenol from aqueous environments, we employed T.E.S.T. software (version 5.1) to predict the toxicity of intermediates identified through HPLC-MS analysis. The results, presented in Figure 8g–j, indicate that most intermediates possess lower toxicity than 2,4-DCP, with several displaying non-toxic characteristics. Furthermore, to enhance the assessment of 2,4-DCP detoxification, we quantified the concentration of Cl^−^ ions in the 2,4-DCP degradation solution through ion chromatography (Figure S7). The findings revealed a significant increase in Cl^−^ ion concentration during the photocatalytic reaction, further supporting the conclusion that 2,4-DCP was being effectively degraded into less toxic compounds.

## 3. Materials and Methods

### 3.1. Chemical Materials

Titanium(IV) isopropoxide (C_12_H_28_O_4_Ti, ≥97.0%), ethylene Glycol (C_2_H_6_O_2_, ≥98%), strontium acetate (C_4_H_6_O_4_Sr, 99.0%), sodium hydroxide (NaOH, 96.0%), cobalt nitrate hexahydrate (Co(NO_3_)_2_·6H_2_O, 99.0%), ethanol (CH_3_CH_2_OH, ≥99.7%), sodium hydroxide (NaOH, ≥96%), peroxymonosulfate (PMS, KHSO_5_·0.5KHSO_4_·0.5K_2_SO_4_, ≥42% KHSO_5_ basis), and 2,4-dichlorophenol (C_6_H_4_Cl_2_O, ≥98%) were purchased from Aladdin Corporation (Shanghai, China). All chemicals were used as received without further purification. Deionized water was utilized to prepare the solutions.

### 3.2. Photocatalyst Preparation

**Scheme 1:**
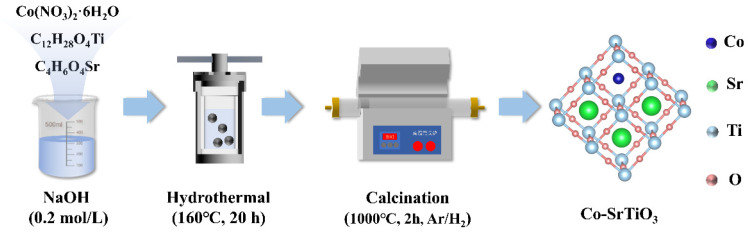
The illustration for the fabrication of Co-SrTiO_3_ composites.

Scheme 1 illustrates the fabrication process for Co-doped SrTiO_3_ composites (Co-SrTiO_3_). Titanium (IV) isopropoxide (0.9 mL) was dissolved in 25 mL of ethylene glycol. Simultaneously, 1.0 g of strontium acetate was dissolved in 25 mL of deionized water. The two solutions were mixed at room temperature and stirred continuously for 30 min. Subsequently, 0.2 g of NaOH and different amounts of Co(NO_3_)_2_·6H_2_O (0.01, 0.025, 0.05, 0.2, and 0.5 g) were added to the suspension, followed by an additional stirring for 30 min. The resulting solution was then transferred to a Teflon-lined stainless-steel autoclave and heated to 160 °C for 20 h. After cooling the mixture to room temperature, the green precipitate obtained by centrifugation was washed three times alternately with deionized water and ethanol. After drying at 80 °C in an oven, the collected powder was transferred to a tubular furnace and calcined at 1000 °C for 2 h under an Ar/H_2_ (95:5) gas mixture. Finally, the resulting black powder was ground to obtain Co-SrTiO_3_. Depending on the amount of Co(NO_3_)_2_·6H_2_O added, the prepared samples were named Co-SrTiO_3_-X (X = 0.01, 0.025, 0.05, 0.2, and 0.5). The preparation process for SrTiO_3_ followed the same steps as for Co-SrTiO_3_, except that Co(NO_3_)_2_·6H_2_O was not added.

### 3.3. Materials Characterization

Phase structures of the samples were analyzed using X-ray powder diffraction (XRD, PANalytical X’Pert3, Malvern Panalytical, Almelo, Netherlands). Sample morphology was examined via scanning electron microscopy (SEM, Hitachi SU8010, Hitachi, Tokyo, Japan) and transmission electron microscopy (TEM, FEI Tecnai F20, Thermo Fisher Scientific, Hillsboro, OR, USA). Chemical states and valence band (VB) edges of the photocatalysts were investigated using a X-ray photoelectron spectrometer (XPS, ESCALAB 250Xi, Thermo Fisher Scientific, Waltham, MA, USA) with Al Kα radiation. UV–Vis diffuse reflectance spectra (UV–Vis DRS) were recorded on a UV–Vis spectrophotometer (U-3900, Hitachi, Tokyo, Japan). Transient-state surface photo voltage measurements were conducted on a CEL-TPV2000 device (Au-Light Co., Beijing, China) using a 355 nm pulsed laser (Q-smart 450, Los Angeles, CA, USA) for excitation. The specific surface area was determined by the Brunauer–Emmett–Teller (BET) method using a Micromeritics ASAP 2020 Plus instrument (Micromeritics, Norcross, GA, USA). Fourier transform infrared (FT-IR) spectra were obtained using a Tensor 27 (Bruker, Billerica, MA, USA). The steady-state photoluminescence (PL) spectra, excited at 350 nm, and the time-resolved PL spectra (TRPL), excited at 375 nm, were analyzed at room temperature using a fluorescence spectrometer (FS5, Edinburgh, Livingston, UK). Photoelectrochemical measurements were performed with a CHI760E electrochemical station (*Chenhua Instruments, Shanghai, China*) using a 300 W Xe lamp. The three-electrode system consisted of samples on the conductive surface of ITO glass as the working electrode, Ag/AgCl as the reference electrode, and Pt wire as the counter electrode, with a 0.25 M Na_2_SO_4_ solution as the electrolyte. The chloride ion concentration in the 2,4-dichlorophenol solution was measured using an ion chromatograph (IC, ICS 1100, Thermo Fisher Scientific, Sunnyvale, CA, USA). Active free radicals involved in photocatalytic reactions were analyzed using electron spin resonance (ESR, A300, Bruker BioSpin, Rheinstetten, Germany. The spin-trapping agent 5,5-dimethyl-1-pyrroline N-oxide (DMPO) was used to detect ·SO_4_^−^ and ·OH radicals in H_2_O and ·O_2_^−^ radicals in methanol. The agents 2,2,6,6-tetramethylpiperidine (TEMP) and 2,2,6,6-tetramethylpiperidine-1-oxyl (TEMPO) were employed to detect ^1^O_2_ non-radicals and photogenerated hole radicals (h^+^), respectively.

### 3.4. Measurement of Photocatalytic Activity

The photocatalytic performance was evaluated by measuring its degradation efficiency towards 2,4-dichlorophenol (2,4-DCP) under visible light irradiation. The photocatalytic reaction was conducted using a photochemical reaction device (CEL-PCRD300-12, Au-light Co., Beijing, China). A 30 mL solution of 2,4-DCP and 0.02 g of the photocatalyst were added to a quartz glass tube and stirred in the dark for 30 min to achieve adsorption–desorption equilibrium. Subsequently, light from a white LED lamp (350 mA, 132 mW·cm^−2^) was introduced along with 0.015 g of PMS. Two milliliter samples were extracted every 10 min. A 0.45 μm filtration membrane was used to separate the 2,4-DCP solution from the solid catalyst. The concentration of 2,4-DCP was determined using a UV–Vis spectrophotometer (λ = 285 nm) [70]. The pollutant removal rate can be calculated using the following Equation (9), where *C_t_* (mg·L^−1^) represents the pollutant concentration at time *t*, and *C*_0_ (mg·L^−1^) denotes the initial concentration.

(9) $$Removal\;rate\;\left(\%\right)=\left(1-C_{t}/C_{0}\right)\times 100\%$$

Intermediate products of 2,4-DCP degradation were identified using LC-MS (Thermo Fisher Vanquish-Q Exactive Plus). Analysis was performed on a Waters ACQUITY BEH C18 column (50 × 2.1 mm, 1.7 µm) at 40 °C. The mobile phase consisted of 0.1% formic acid and acetonitrile with a flow rate of 0.3 mL/min, following gradient: 5% acetonitrile (0–1 min), 95% acetonitrile (1–7 min), 95% acetonitrile (7–10 min), and 5% acetonitrile (12–15 min). UV detection was at 214 nm and 254 nm. The ESI source operated in negative ion mode with a capillary voltage of 3000 V. The injection volume was 5 µL.

### 3.5. Theory Calculations

Density functional theory (DFT) calculations, which included the electron localization function, Bader charges, and adsorption energies, were conducted using the Vienna Ab initio Simulation Package (VASP) version 5.4.4 [71]. The generalized gradient approximation (GGA) with the Perdew–Burke–Ernzerhof (PBE) exchange-correlation functional was utilized [72]. Self-consistent electronic calculations were performed with a plane-wave cutoff energy set to 450 eV. The Fukui function values were calculated using the Gaussian 16 program at the B3LYP/6-31G* level of theory [73,74]. Additionally, the Hirshfeld charge was computed with the Multiwfn 3.8 program [75].

## 4. Conclusions

In conclusion, this study successfully enhanced the degradation activity of SrTiO_3_ in 2,4-DCPs through cobalt doping, which primarily attributed to the following aspects: (1) Adjusting the metal (Ti) d-band center to improve the PMS activation capability and the charge transfer ability to PMS; (2) optimizing the energy band structure and work function of SrTiO_3_, thereby enhancing solar light utilization and carrier extraction capability; (3) modifying the surface morphology of SrTiO_3_ to increase the specific surface area and the number of active sites; and (4) broadening the pH response range and improving the stability of the catalyst, making it possible to maintain catalytic activity under different water quality conditions, and thus, holding broad application prospects. This research not only provides new catalyst design and optimization ideas for PMS-based advanced oxidation technology but also offers an efficient and environmentally friendly solution for treating refractory organic pollutants in industrial wastewater.

## Data Availability

The data presented in this study are available on request from the corresponding author.

## Supplementary Materials

The following supporting information can be downloaded at: https://www.mdpi.com/article/10.3390/molecules30122618/s1. Figure S1: SEM images of SrTiO_3_ and Co-SrTiO_3_; Figure S2: (a) N2 adsorption-desorption characteristics, (b) BJH pore size distribution plots of SrTiO_3_ and Co-SrTiO_3_-0.2; Figure S3: Energy Dispersive Spectroscopy (EDS) of Co-SrTiO_3_-0.2; Figure S4: (a) Cyclic photodegradation with PMS activation degradation of 2,4-DCP (30 mL,30 mg/L) on the Co-SrTiO_3_-0.2 composite. (b) The XRD patterns of Co-SrTiO_3_-0.2 before and after fifth photocatalytic cycling reaction; Figure S5: ESR spectrum of surface oxygen vacancies on SrTiO_3_ and Co-SrTiO_3_; Figure S6: The liquid chromatography-tandem mass spectrometry intermediate signal of 2,4-DCP degradation solution over Co-SrTiO_3_ after 10 min of light radiation; Figure S7: Plots of Cl- concentration of photocatalytic 2,4-DCP degradation solution versus reaction time; Table S1: The results of SrTiO_3_ and Co-SrTiO_3_-0.2 obtained from N2 adsorption-desorption isotherms; Table S2: Fitted parameters for the time-resolved PL decay curves; Table S3: Fukui function values for 2,4-DCP.
