# Supplementary material for: Modulating D-Band Center of SrTiO3 by Co Doping for Boosted Peroxymonosulfate (PMS) Activation Under Visible Light
Source: Molecules. 2025 Jun 17;30(12):2618. doi: 10.3390/molecules30122618 (PMC12196530; doi:10.3390/molecules30122618)
Supplement: Supplementary file 1 [file molecules-30-02618-s001.zip › molecules-3691125-supplementary.pdf]

Supplementary information for

Modulating d-Band Center of $SrTiO_3$ by Co doping for boosted Peroxymonosulfate (PMS) activation under visible light

Kaining Sun[a], Xinyi Yang[a], Fei Qi[b], Yingjie Liu[b], Lijing Wang[c], Bo Feng[b,*], Jiankang Yu[a,*], Guangbo Che[b,*]

[a] *College of Safety Science and Engineering, Liaoning Technical University, Huludao 125105, PR China.*

[b] *Jilin Provincial Key Laboratory of Western Jilin's Clean Energy, Baicheng Normal University, Baicheng 137000, PR China*

[c]*Henan Engineering Center of New Energy Battery Materials, Henan D&A Engineering Center of Advanced Battery Materials, College of Chemistry and Chemical Engineering, Shangqiu Normal University, Shangqiu 476000, P. R. China*

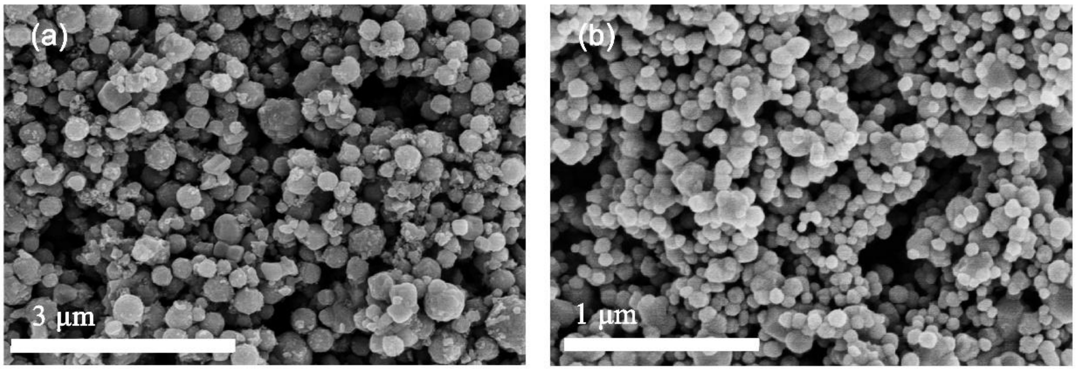

Figure S1. SEM images of $SrTiO_3$ and Co-$SrTiO_3$.

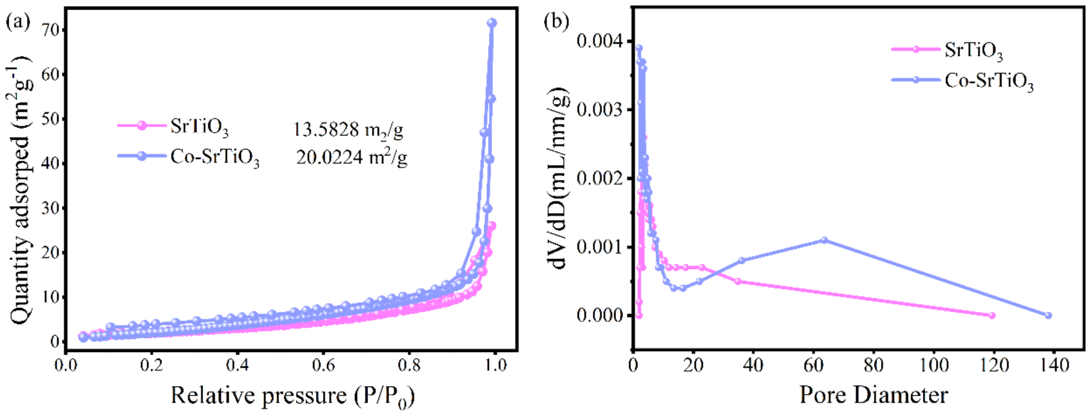

Figure S2. (a) N_2 adsorption-desorption characteristics, (b) BJH pore size distribution plots of $SrTiO_3$ and Co-$SrTiO_3$-0.2.

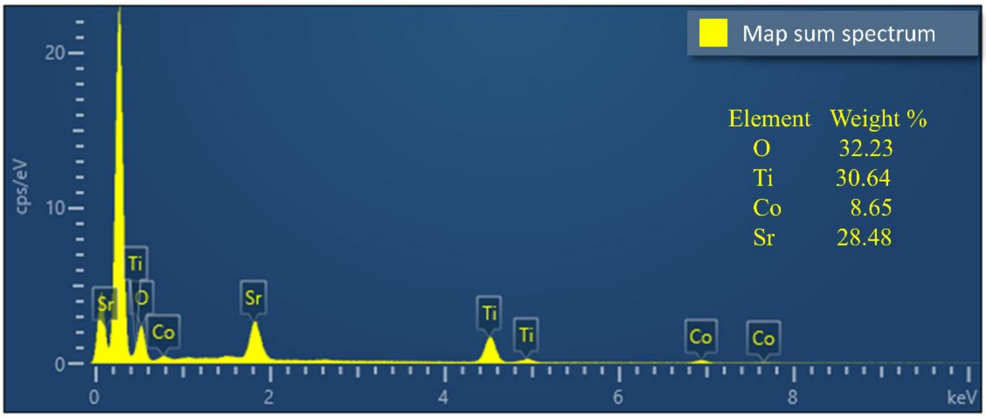

Figure S3: Energy Dispersive Spectroscopy (EDS) of Co-$SrTiO_3$-0.2.

Table S1. The results of $SrTiO_3$ and Co-$SrTiO_3$-0.2 obtained from N_2 adsorption-desorption isotherms.

Sample	S_{BET}（$m^2 \cdot g^{-1}$）	Pore size (nm)	Pore volume ($cm^3 \cdot g^{-1}$)
$SrTiO_3$	13.5828	19.8977	0.0396
Co-$SrTiO_3$-0.2	20.0224	36.6590	0.0914

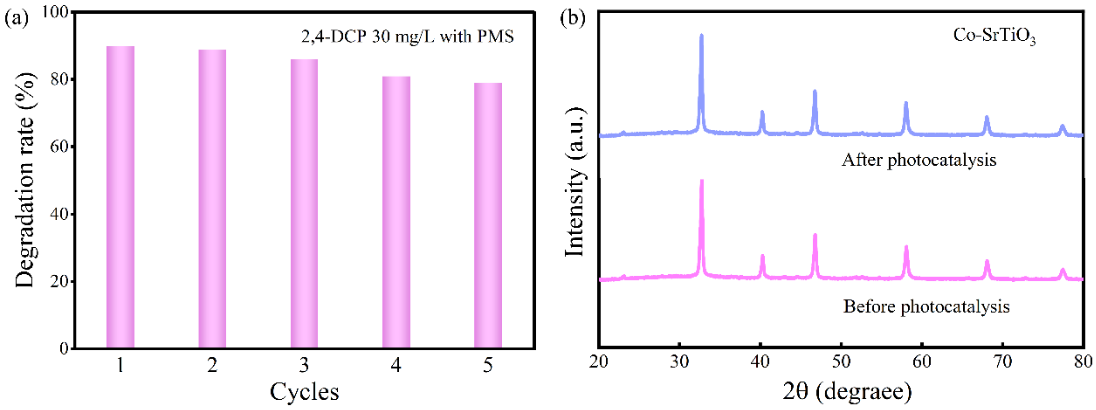

Figure S4. (a) Cyclic photodegradation with PMS activation degradation of 2,4-DCP (30 mL, 30 mg/L) on the Co-$SrTiO_3$-0.2 composite. (b) The XRD patterns of Co-$SrTiO_3$-0.2 before and after fifth photocatalytic cycling reaction.

Table S2. Fitted parameters for the time-resolved PL decay curves.

Photocatalyst	A_1	τ_1	A_2	τ_2	τ_A
$SrTiO_3$	1.39065	0.58787	37.77086	5.83431	0.58787
Co-$SrTiO_3$	206.58453	4.26049	1.21165	19.64042	4.66538

The following equation was used to calculate the average PL lifetime (τ_A):

$$\tau_A = (A_1\tau_1^2 + A_2\tau_2^2)/(A_1\tau_1 + A_2\tau_2)$$

where A_1 and A_2 represented the relative amplitudes of decay factors, and τ_1 and τ_2 standed for the decay times, individually.

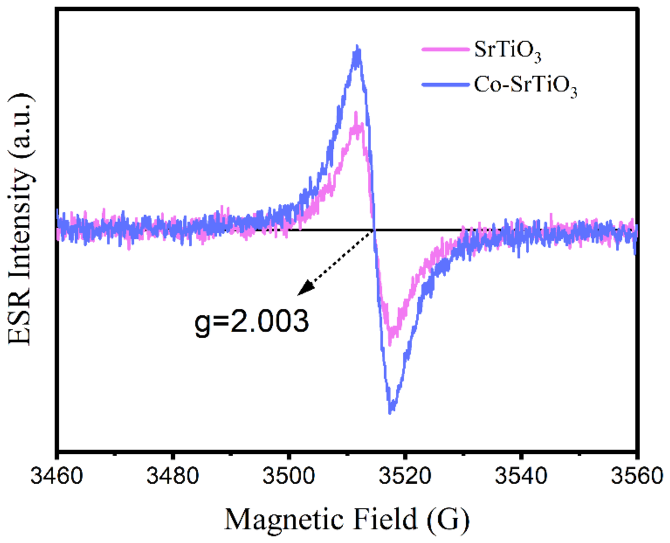

Figure S5. ESR spectrum of surface oxygen vacancies on $SrTiO_3$ and Co-$SrTiO_3$.

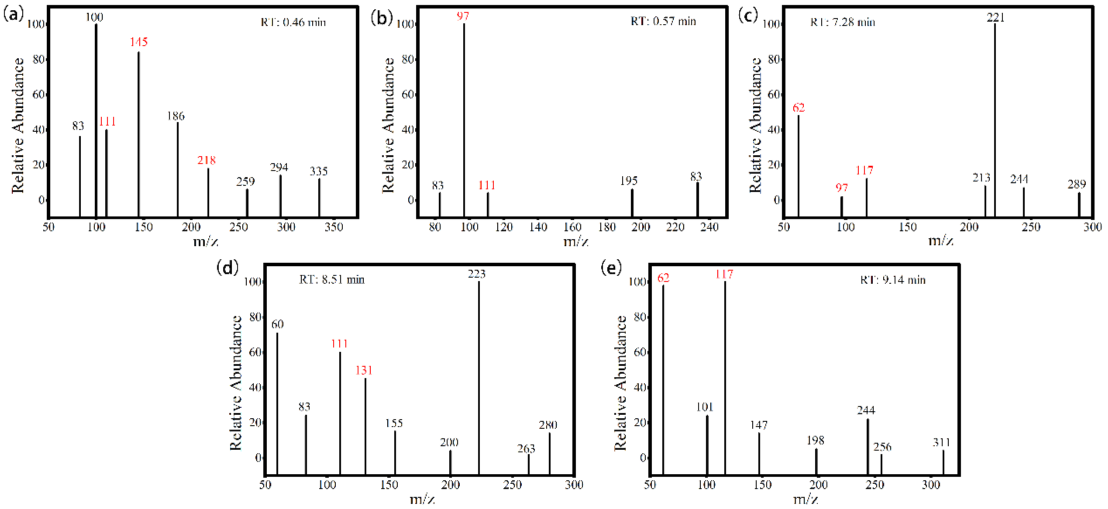

Figure S6. The liquid chromatography-tandem mass spectrometry intermediate signal of 2,4-DCP degradation solution over Co-$SrTiO_3$ after 10 min of light radiation.

Table S3. Fukui function values for 2,4-DCP

Atom	q(N)	q(N+1)	q(N-1)	f^-	f^+	f^0
1 (C)	-0.0554	-0.1755	0.0012	0.0566	0.1201	0.0884
2 (C)	-0.0395	-0.1559	0.0324	0.0719	0.1164	0.0942
3 (C)	0.0235	-0.0140	0.1054	0.0818	0.0375	0.0597
4 (C)	-0.0419	-0.1615	-0.0029	0.0390	0.1196	0.0793
5 (C)	0.0197	-0.0759	0.0801	0.0604	0.0956	0.0780
6 (C)	0.0746	0.0319	0.1582	0.0836	0.0427	0.0632
7 (O)	-0.1887	-0.2291	-0.0744	0.1143	0.0403	0.0773
8 (Cl)	-0.0657	-0.1978	0.0882	0.1539	0.1321	0.1430
9 (Cl)	-0.0833	-0.1854	0.1054	0.1887	0.1020	0.1454
10 (H)	0.0508	-0.0055	0.0878	0.0370	0.0563	0.0467
11 (H)	0.0569	0.0017	0.0953	0.0384	0.0552	0.0468
12 (H)	0.0604	0.0065	0.0914	0.0310	0.0539	0.0424
13 (H)	0.1888	0.1607	0.2319	0.0432	0.0281	0.0356

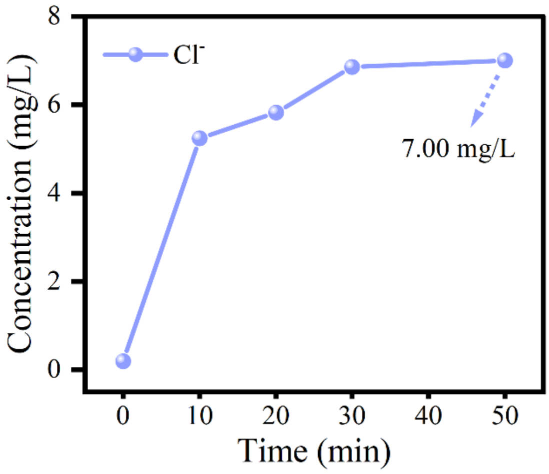

Figure S7. Plots of Cl^- concentration of photocatalytic 2,4-DCP degradation solution versus reaction time.
